# Experimental Investigation of Moisture Sensitivity and Damage Evolution of Porous Asphalt Mixtures

**DOI:** 10.3390/ma14237151

**Published:** 2021-11-24

**Authors:** Xinyu Hu, Xiaowei Wang, Nanxiang Zheng, Qiang Li, Jinyue Shi

**Affiliations:** 1 Key Laboratory of Special Area Highway Engineering of Ministry of Education, Chang’an University, Xi’an 710064, China; xinyu_hu2021@163.com (X.H.); emailznx@163.com (N.Z.); 2 School of Civil Engineering, Xi’an University of Architecture and Technology, Xi’an 710055, China; 3 School of Civil Engineering, Nanjing Forestry University, Nanjing 210037, China; liqiang2526@njfu.edu.cn; 4 Chongqing Changshou District Highway Affairs Center, Chongqing 401220, China; baboy90@163.com

**Keywords:** porous asphalt mixtures, moisture sensitivity, moisture damage, damage evolution, durability

## Abstract

Porous asphalt (PA) mixtures are designed with a high air void (AV) (i.e., 18~22%) content allowing rainwater to infiltrate into their internal structures. Therefore, PA mixtures are more sensitive to moisture damage than traditional densely graded asphalt mixtures. However, the moisture damage evolution of PA mixtures is still unclear. The objective of this study was to investigate the moisture damage evolution and durability damage evolution of PA mixtures. The indirect tensile test (ITT), ITT fatigue test, and Cantabro loss test were used to evaluate the moisture sensitivity and durability of PA mixtures, and a staged ITT fatigue test was developed to investigate the damage evolutions under dry and wet conditions. Indirect tensile strength (ITS), fatigue life, indirect tensile resilience modulus (E), and durability decreased with the increment of moisture damage and loading cycles. The fatigue life is more sensitive to the moisture damage. The largest decrements in ITS and E were found in the first 3000 loading cycles, and PA mixtures tended to fail when the decrement exceeded 60%. Damage factors based on the ITS and E are proposed to predict the loading history of PA mixtures. The durability damage evolution and damage factors could fit an exponential model under dry conditions. Moisture had a significant influence and an acceleration function on the moisture damage evolution and durability damage evolution of PA mixtures.

## 1. Introduction

Porous asphalt (PA) mixtures are formed by an open graded asphalt mixture with a high air void (AV) content (i.e., 18~22%) and an open graded skeleton. PA mixtures have the benefits of improving drive safety, mitigation of spray and hydroplaning, and noise reduction [1,2]. Due to the frequent and direct exposure to water, PA mixtures are more sensitive to moisture damage, and the distresses of raveling and stripping caused by moisture damage are the main concerns during the application of PA mixtures [3,4,5]. Therefore, evaluating the moisture sensitivity and understanding moisture damage evolution is of great significance for the design of PA mixtures.

There are two categories of moisture sensitivity tests: one is performed on loose asphalt mixtures and the other is conducted on compacted asphalt mixtures [6,7]. Static immersion tests (AASHTO T182, ASTM D1664) [6,8], boiling water test (ASTM D3625) [9], and dynamic immersion tests (WHI 90) [6] are the main tests performed on the loose asphalt mixtures. These tests mainly involve coating aggregates with asphalt and being immersed in water in a specified condition. Then, the loss percentage of asphalt stripped off the aggregate was estimated and used to evaluate the moisture sensitivity. However, studies have indicated that these test results are subjective when using a visual evaluation. The surface energy theory test is a quantitative test used to characterize the moisture damage of loose asphalt mixtures, and the adhesive bond energy between asphalt and aggregate were calculated [10,11]. In addition, Wang et al. [3] developed a binder bond strength (BBS) test and modified BBS test to measure the adhesion and cohesion of asphalt, mastic, and mortar in PA mixtures. The adhesion and cohesion can be used to evaluate the moisture sensitivity of PA mixtures.

Moisture sensitivity tests for compacted asphalt mixtures involve a mechanical evaluation of specimens before and after moisture damage. The modified Lottman test (AASHTO T-283) [12], the indirect tensile test (ITT), and the immersion Marshall stability test are common moisture sensitivity tests for compacted asphalt mixtures [13,14,15,16,17,18,19]. The dynamic modulus test was performed to evaluate the moisture sensitivity of PA mixtures under dry and wet conditions [20]. Furthermore, to better simulate the field moisture condition, the immersion wheel tracking test and Hamburg wheel tracking test were designed to test the asphalt mixtures in a water condition [6,21]. To simulate the pumping action, a Moisture-Induced Sensitivity Tester (MIST) was designed to keep samples in a constant temperature and pressure [14,22]. Among them, the ITT is the most widely used test for evaluating the moisture sensitivity of PA mixtures [16,23]. The indirect tensile strength (ITS) obtained from the ITT was compared in dry and wet conditions, and the tensile strength ratio (TSR) of dry to wet was calculated to evaluate the potential moisture damage. Poulikakos and Partl developed the coaxial shear test (CAST) to simulate field conditions and evaluate the moisture sensitivity of PA mixtures based on a fatigue test [24,25]. The results indicated that the effect of water immersion on PA mixtures is more pronounced than on dense-graded asphalt mixtures.

Moisture damage plays an important role in the raveling of PA mixtures. Mo et al. established a 2D and 3D Finite Element model to analyze the adhesion failure between aggregate and bitumen [26,27]. Caro et al. developed a Finite Element model with Cohesive Zone Elements to quantify the potential initiation of raveling of PA mixtures [28]. Zhang et al. used 3D-MOVE tools and a homogenization technique to compute the stress and strain in PA mixtures and analyze the raveling distress [29]. However, the moisture was not taken into consideration in the numerical modeling. In addition, previous studies mainly evaluated the moisture damage of PA mixtures by comparing the mechanical properties before and after moisture sensitivity tests. The moisture damage evolution of PA mixtures has not been experimentally elaborated, both in laboratory tests and numerical modeling.

The objective of this study was to investigate moisture sensitivity, moisture damage evolution, and durability damage evolution of PA mixtures using laboratory tests. The ITT and ITT fatigue test were applied to evaluate the moisture sensitivity of PA mixtures under different wet conditions. A staged ITT fatigue test was developed to characterize the moisture damage evolution of PA mixtures. Based on the results, the relationships between ITS, indirect tensile resilience modulus, loading cycles, and durability are discussed in the moisture damage process.

## 2. Materials

**Figure 1:**
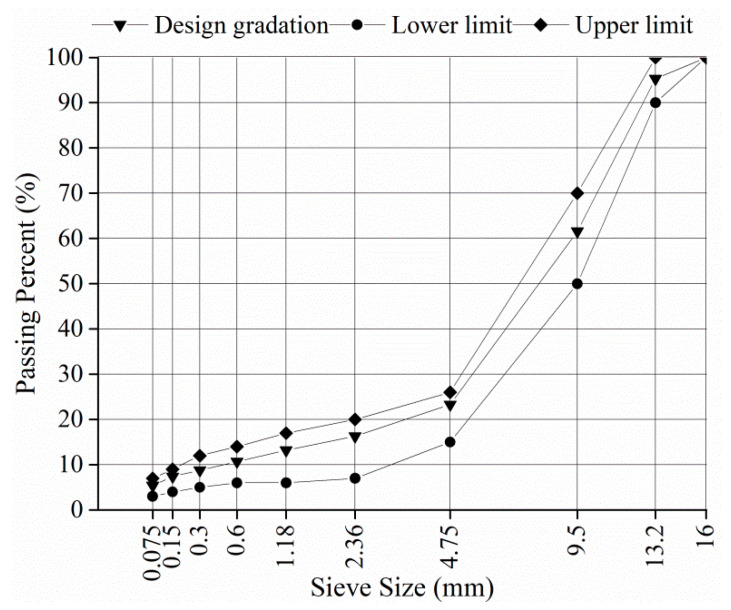
Gradation of PA-13.

The PA mixtures used in the surface layer in China usually had a nominal maximum aggregate size (NMAS) of 13.2 mm. A typical PA mixture with an NMAS of 13.2 mm (PA-13) was investigated in this study. The gradation of PA-13 is shown in Figure 1. A high viscosity modified asphalt (HVA) produced by SK energy company was used for PA-13. The properties of HVA are listed in Table 1. Basalt was used for the coarse and fine aggregates of PA-13. The optimum asphalt content is 4.8%, and the target AV content is 20%.

Specimens were compacted by a Superpave gyratory compactor with 50 gyrations to obtain the target AV content [30,31]. The height of the specimen is 63.5 mm, and the diameter is 100 mm. At least three replicates were prepared for the ITT, Cantabro loss test, and ITT fatigue test, and six replicated specimens were prepared for the staged ITT fatigue test.

## 3. Methods

In this study, ITT and ITT fatigue tests were used to evaluate the moisture sensibility, and the Cantabro loss test was performed to evaluate the durability of PA mixtures under dry and wet conditions. In addition, a staged ITT fatigue test was developed to investigate the damage evolutions of PA mixtures under dry and wet conditions.

### 3.1. ITT

ITT was performed on universal testing machine (UTM, IPC Global, Melbourne, Victoria, Austrilia), and a diametral line load was applied on specimens with a displacement of 50 mm/min until the maximum failure strength was reached. The ITS was calculated based on Equation (1). ITT was performed under dry and wet conditions, and TSR was calculated with Equation (2). For the dry condition, the specimens were placed in a thermostatic chamber with a constant temperature of 20 °C for 4 h prior to testing. For the wet conditions, the specimens were in a water bath at a constant temperature of 60 °C for 2 days (2 d), 4 days (4 d), 6 days (6 d), and 8 days (8 d). Then, the specimens were placed in a water bath at 20 °C for 2 h prior to testing. China specified a wet condition that specimens were immersed in a 60 °C water bath for two days to evaluate the moisture sensitivity of asphalt mixtures [32]. In this paper, wet conditions of 4 d, 6 d, and 8 d with more serious moisture damage were evaluated to investigate the long-term moisture sensitivity of PA mixtures.

(1) $$ITS=\frac{2F}{\pi hD}$$

where *F* is the maximum failure load, kN; *h* is the height of the specimen, mm; and *D* is the diameter of the specimen, mm.

(2) $$TSR=\frac{ITS_{dry}}{ITS_{wet}},$$

where *ITS_dry_* is the *ITT* strength under dry condition, kPa; *ITS_wet_* is the ITT strength under wet condition, kPa.

### 3.2. ITT Fatigue Test

**Figure 2:**
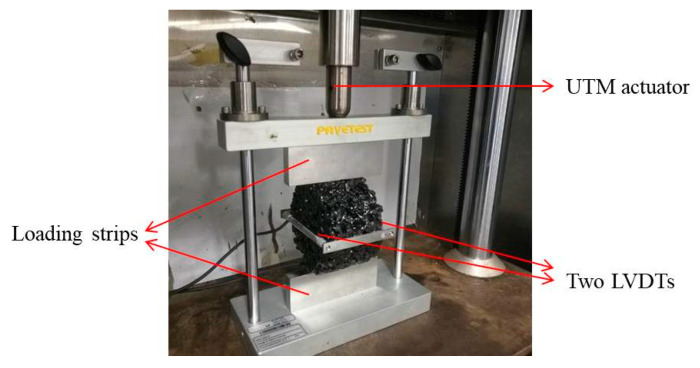
ITT fatigue test.

The ITT fatigue test was used to evaluate the cracking resistance and moisture sensitivity of asphalt mixtures [33,34]. Cracking was mainly caused by the loss of adhesion between aggregates and asphalt or loss of cohesion within asphalt [35,36]. It has the same failure mechanism as with moisture damage. Therefore, the ITT fatigue test was used to evaluate the moisture sensitivity of PA mixtures. As shown in Figure 2, specimens were located between two loading strips with a concave surface, and two linear variable differential transducers (LVDTs) were glued on the opposite sides of the horizontal diametral plan. The horizontal deformation was measured by the LVDTs, and the horizontal permanent strain (*ε*) was calculated based on Equation (3) when Poisson’s ratio was 0.35. The initial indirect tensile resilience modulus (*E_0_*) was defined as the indirect tensile resilience modulus (*E*) at 100 loading cycles and calculated by Equation (4).

(3) $$\varepsilon =2.1\frac{\Delta H}{D}$$

where Δ*H* is the horizontal deformation, mm.

(4) $$E_{0}=\frac{1000P_{0}(1+3\nu )}{\varepsilon_{100}}$$

where *P_0_* is the stress levels used in the ITT fatigue test, MPa; *υ* is the Poisson’s ratio; and *ε_100_* is the horizontal permanent strain at the loading cycle number of 100.

There are two loading models for the ITT fatigue test. One is constant displacement mode, where a cyclic constant displacement was applied on specimens until the specimens failed. The other is constant loading mode, where a cyclic constant load was applied on the specimens. The constant loading mode was selected in this paper because the vehicle loading applied on the pavement was repetitive and the deformation was nonlinear. Haversine cyclic loading was applied at a frequency of 10 Hz (0.1 s loading cycle) without a rest period. Several stress levels corresponding to 0.2, 0.25, and 0.3 of the ITS were selected to be applied on the specimens. The ITT fatigue test was performed under the dry and wet conditions (2 d, 4 d, 6 d, and 8 d), and the test temperature was 20 °C. Fatigue life corresponded to the number of cycles when specimens completely failed.

### 3.3. Staged ITT Fatigue Test

To investigate the moisture damage evolution of PA mixtures, a staged ITT fatigue test was developed. The ITT fatigue test was applied for 3000 cycles, 6000 cycles, 9000 cycles, and 12,000 cycles under dry and wet conditions (2 d), respectively. Six replicated specimens were prepared for each test, and the test program is shown in Table 2. After the staged ITT fatigue test, ITT and Cantabro loss tests were performed on the tested specimens. The six replicated specimens include three specimens for the ITT and three specimens for the Cantabro loss test. Before the staged ITT fatigue test, the location of the loading strips on the specimen was marked and used for the following ITT. The residual ITS, E, and durability were used to investigate the moisture damage evolution of PA mixtures.

### 3.4. Cantabro Loss Test

The Cantabro loss test has been recommended to evaluate the durability of PA mixtures and is used in many countries [27,34,35]. The Cantabro loss test involves placing a specimen into a Los Angeles (LA) abrasion machine with 300 revolutions. As shown in Equation (5), the Cantabro loss is calculated based on the mass of the specimen before and after the test.

(5) $$C\mathrm{antabro}\;\mathrm{loss}=\frac{m_{0}-m_{1}}{m_{0}}\times 100\%$$

where *m_0_* is the mass before the test, g; *m_1_* is the mass after the test, g.

## 4. Results

### 4.1. Determination of the Optimum Stress Ratio for ITT Fatigue Test

**Figure 3:**
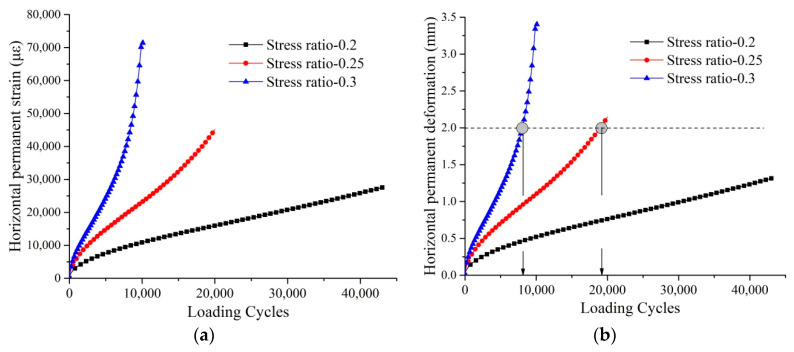

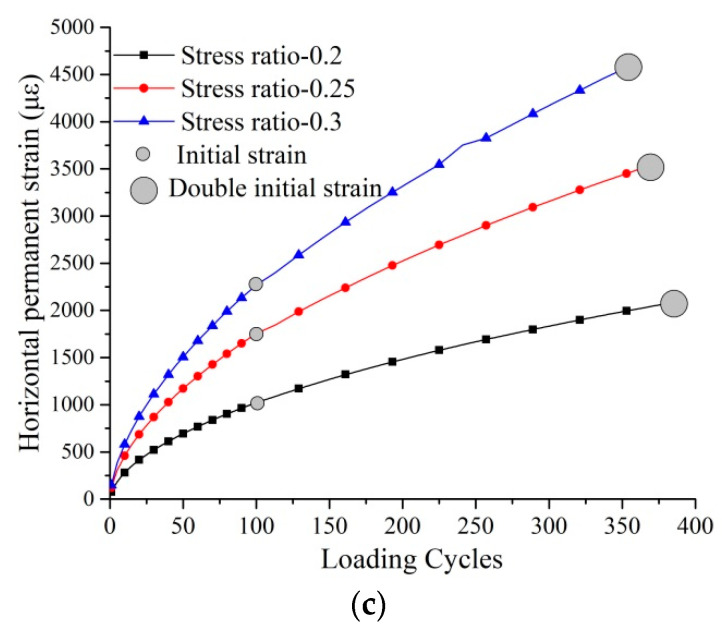
Horizontal permanent deformation under different stress ratios. (**a**) Horizontal permanent strain; (**b**) horizontal permanent deformation; (**c**) horizontal permanent strain at the start of the ITT fatigue test.

Based on the ITT, the average ITS under the dry condition was measured as 872 kPa. The stress ratio is defined as the ratio of stress levels used in the ITT fatigue test to the ITS. Figure 3a,b shows the horizontal permanent strain and horizontal permanent deformation against the loading cycles under the stress ratio of 0.2, 0.25, and 0.3 under the dry condition. Under the stress ratio of 0.3, the horizontal deformation developed the fastest, and the specimens failed the earliest. Regarding the failure criteria, the determination of the actual point of fatigue failure is a controversial topic with various definitions involving phenomenological as well as energy approaches [33]. In addition, the failure criteria used for dense-graded asphalt mixtures may not suitable for the PA mixtures. In the European standard (EN 12697-24), the failure criteria are defined as the displacement having increased to double that of the initial strain of the test under the constant loading mode [37]. The initial strain is the horizontal permanent strain at the 100th loading cycle. Figure 3c shows the horizontal permanent stain at the start of the ITT fatigue test. The initial stain was 1023 με, 1755 με, and 2271 με under the stress ratio of 0.2, 0.25, and 0.3, respectively. As shown in Figure 3c, when the loading cycles were reached at the 385, 369, and 353 loading cycles under the stress ratio of 0.2, 0.25, and 0.3, respectively, the horizontal permanent stain increased by double that of the initial strain. However, the specimens were not cracked under that failure criterion. Both horizontal deformation and the failure life are too small to distinguish the differences between different stress ratios. A higher horizontal permanent deformation is needed in the failure criteria for PA mixtures because the higher AV content allows higher deformation to happen. In this study, the failure criteria used for the ITT fatigue test is when the horizontal permanent deformation reached 2 mm, and this was determined by observing the tested specimens when they were completely fractured. Therefore, the failure life under the stress ratio of 0.25 and 0.3 were 19,072 and 8049, respectively, as shown in Figure 3b. Specimens did not show fractures under the stress ratio of 0.2 even after 43,000 loading cycles. Considering the time efficiency and discrimination between different conditions, the stress ratio of 0.25 was determined as the optimum stress ratio for the ITT fatigue test.

### 4.2. Evaluation of the Moisture Sensitivity of the PA Mixture

**Figure 4:**
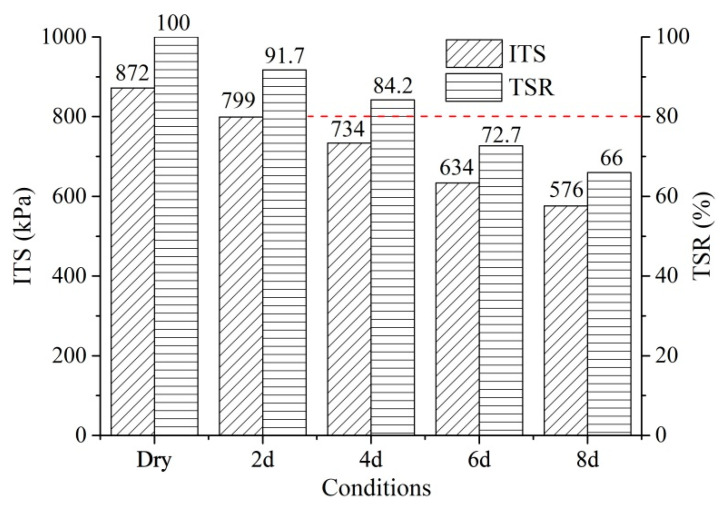
ITS under different conditions.

Figure 4 shows the ITS under dry and wet conditions. It has been recognized that the moisture damage increased with the increasing immersion time in a water bath. It can be found that the ITS decreased with the increment of moisture damage. Compared with the dry condition, the ITS decreased by 8.3%, 15.8%, 27.3%, and 34.0% under the wet conditions of 2 d, 4 d, 6 d, and 8 d, respectively. The PA mixtures showed a good moisture resistance under the wet conditions of 2 d. However, the PA mixtures under the wet conditions of 6 d and 8 d could not satisfy the requirement that TSR should be greater than 80%. The long-term moisture sensitivity of PA mixtures is still a concern in their service life.

**Figure 5:**
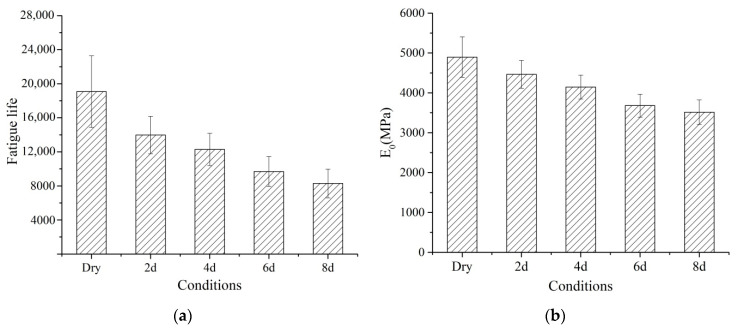
Fatigue life and E_0_ under different conditions: (**a**) fatigue life; (**b**) E_0_.

Figure 5a,b shows the fatigue life and E_0_ under the dry and wet conditions, respectively. Fatigue life significantly decreased with the increment in moisture damage. The fatigue life of the dry, 2 d, 4 d, 6 d, and 8 d is 19,072, 13,984, 12,288, 9696, and 8288, respectively. E_0_ also decreased with the increment in moisture damage. The E_0_ of the dry, 2 d, 4 d, 6 d, and 8 d is 4897 MPa, 4466 MPa, 4147 MPa, 3680 MPa, and 3516 MPa, respectively. Therefore, the fatigue life and E_0_ can differentiate the moisture sensitivity of PA mixtures.

**Figure 6:**
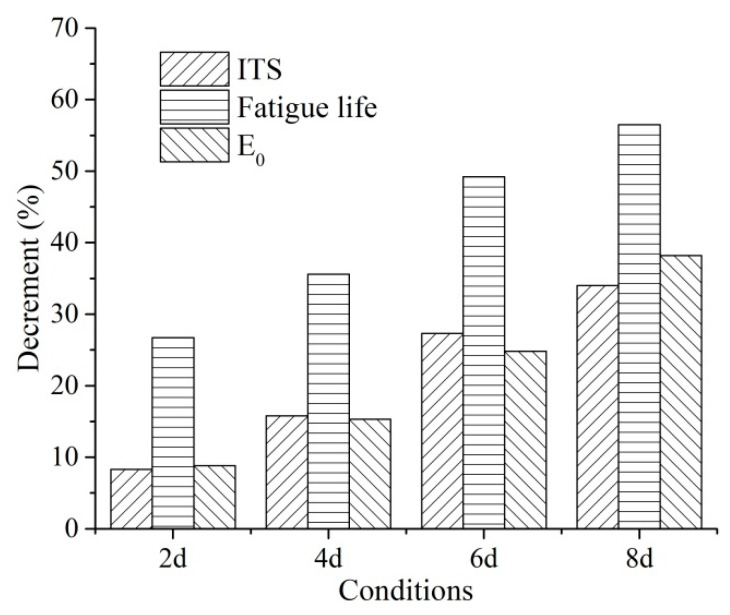
Decrement in ITS, fatigue life, and E0 compared with dry conditions.

Figure 6 shows the decrement in ITS, fatigue life, and E_0_ compared with the dry conditions. Compared with the dry conditions, the fatigue life decreased by 26.7%, 35.6%, 49.2%, and 56.5% under the wet conditions of 2 d, 4 d, 6 d, and 8 d, respectively. There was a great decrease in fatigue life under the 2 d condition. Compared with the dry condition, E_0_ decreased by 8.8%, 15.3%, 24.8%, and 28.2% under the wet conditions of 2 d, 4 d, 6 d, and 8 d, respectively. As shown in Figure 6, under the same condition, the decrement in fatigue life was the largest. This indicates that fatigue life is more sensitive to moisture damage than ITS and E_0_.

### 4.3. Moisture Damage Evolution of PA Mixture

**Figure 7:**
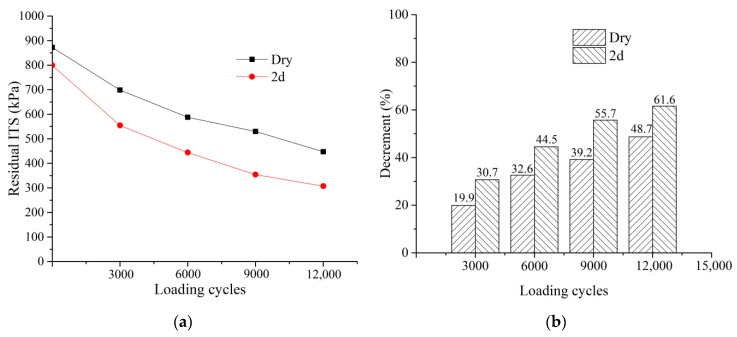
Degradation of ITS: (**a**) residual ITS against the loading cycles; (**b**) decrement in ITS.

Moisture damage evolution under the dry and 2 d conditions was investigated. ITT was performed after the staged ITT fatigue test, and the residual ITS was obtained. E was calculated after the staged ITT fatigue test and compared with the E_0_. Figure 7a shows the residual ITS at different loading cycles. The residual ITS under the dry condition was greater than 2 d through the fatigue life, indicating that moisture damage had a significant influence on the damage evolution of ITS. Figure 7b shows the decrement of residual ITS of each stage when compared with the original. Overall, the decrement in ITS increased with the loading cycles, and the decrement under the 2 d condition were greater than the dry condition. Compared with the previous stage, the decrements in ITS of 3000, 6000, 9000, and 12,000 loading cycles are 19.9%, 12.7%, 6.6%, and 9.5%, respectively, under the dry conditions. Compared with the previous stage, the decrements in ITS of 3000, 6000, 9000, and 12,000 loading cycles are 30.7%, 13.8%, 11.2%, and 5.9%, respectively, under the 2 d conditions. The decrement in ITS in the first 3000 loading cycles is the largest under the dry and 2 d conditions. Therefore, the ITS of the PA mixtures mainly decreased in the early stage.

**Figure 8:**
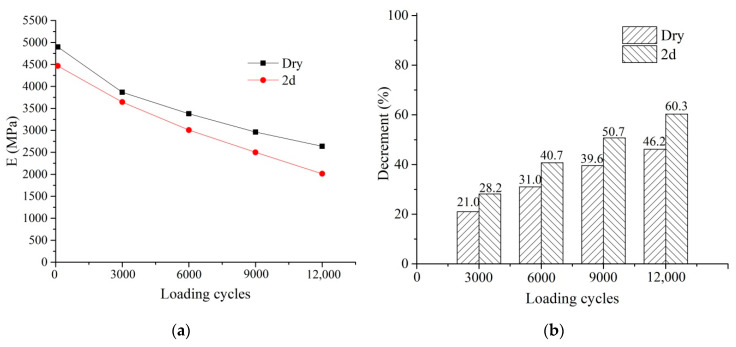
Degradation of E: (**a**) E against the loading cycles; (**b**) decrement in E.

Figure 8a shows E at different loading cycles under the dry and 2 d conditions. The E decreased with the loading cycles, and the E under the dry condition was greater than the 2 d through the fatigue life. Figure 8b shows the decrement in E at different stages compared to the original. The largest decrement was also found in the first 3000 loading cycles, and the decrement under 2 d was greater than that in the dry condition. The specimens failed at 13,984 loading cycles under the 2 d condition, and the ITS and E were decreased by more than 60% after 12,000 cycles. It can be concluded that the specimens tended to fail when the decrement in ITS and E exceeded 60%.

### 4.4. Durability Damage Evolution of PA Mixture

**Figure 9:**
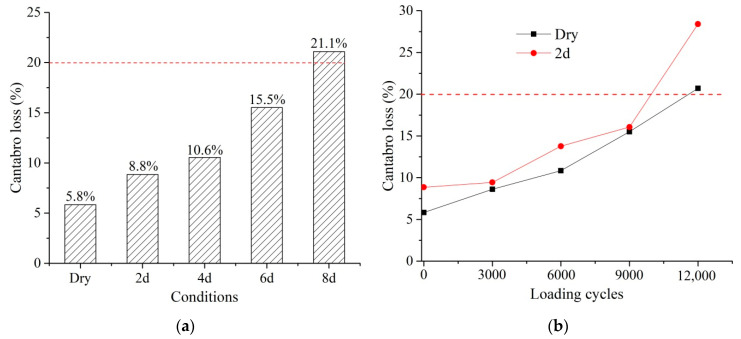
Cantabro loss test: (**a**) Cantabro loss under dry and wet conditions; (**b**) Cantabro loss at different loading cycles.

The Cantabro loss test was performed under the dry and wet conditions, as well as after the staged ITT fatigue test. Figure 9a presents the Cantabro loss under the dry and wet conditions. The Cantabro loss increased with the increment in moisture damage. The results indicate that moisture damage had a significant effect on the durability of PA mixtures. The Cantabro loss after 8 d was greater than 20%, which is the maximum recommended by many researchers and specifications [27,35,37,38], while other conditions have a value below 20%.

Figure 9b shows the Cantabro loss at different loading cycles. The Cantabro loss under the dry condition was lower than that of 2 d through the fatigue life. At the loading cycles of 12,000, specimens tended to fail, showing a great increment in Cantabro loss. After 12,000 loading cycles, the Cantabro loss of PA mixtures did not satisfy the requirement that the Cantabro loss should be smaller than 20%.

## 5. Discussion

**Figure 10:**
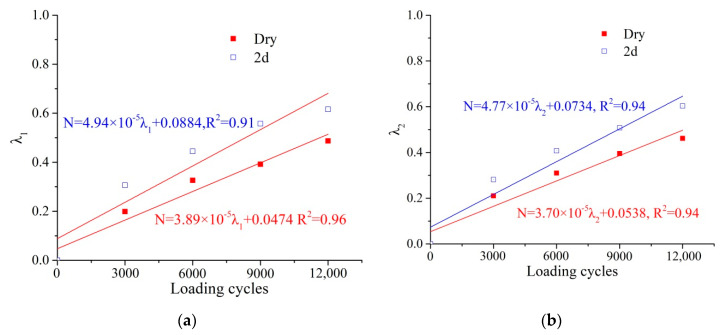
Relationship between damage factors and loading cycles. (**a**) *λ*_1_ and loading cycles; (**b**) *λ*_2_ and loading cycles.

As shown in Equations (6) and (7), two damage factors (*λ_1_* and *λ_2_*) were proposed to investigate the damage evolution of PA mixtures. The relationships between the damage factors and loading cycles are shown in Figure 10a,b.

(6) $$\lambda_{1}=1-\frac{ITS_{i}}{ITS_{0}}$$

(7) $$\lambda_{2}=1-\frac{E_{i}}{E_{0}},$$

where *ITS_i_* is the *ITS* at the loading cycles of *i* (3000, 6000, 9000, and 12,000), kPa; *ITS_0_* is the *ITS* without loading history, kPa; and *E_i_* is the *E* at the loading cycles of *i* (3000, 6000, 9000, and 12,000), MPa.

A fitting analysis was conducted between the damage factors and loading cycles. As shown in Figure 10a,b, *λ_1_* and *λ_2_* presented a good linear relationship to the loading cycles, and the correlation coefficient is greater than 0.9. This indicates that the loading history that was applied on the PA mixtures can be predicted based on the damage factors.

**Figure 11:**
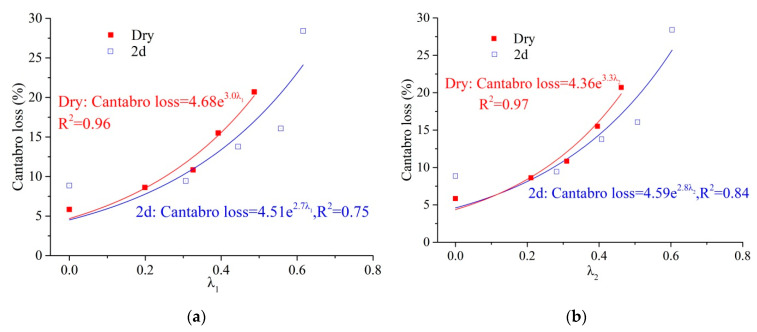
Relationships between damage factors and Cantabro loss. (**a**) *λ*_1_ and Cantabro loss; (**b**) *λ*_2_ and Cantabro loss.

Figure 11a shows the relationship between *λ_1_* and the Cantabro loss, and Figure 11b shows the relationship between *λ_2_* and the Cantabro loss during the damage evolution. Under the dry condition, the results indicate that the Cantabro loss and damage factors could fit an exponential model well, and the correlation coefficient is greater than 0.95. The durability damage evolution correlated well with the damage factors. However, under the 2 d condition, the Cantabro loss and damage factors could not fit well with the exponential model. The damage evolution of durability is more complicated under the effects of moisture damage.

## 6. Conclusions

In this paper, the ITT, ITT fatigue test, staged ITT fatigue test, and Cantabro loss test were used to evaluate the moisture sensitivity, moisture damage evolution, and durability damage evolution of PA mixtures. Several conclusions can be drawn as follows:

(1) ITS, fatigue life, and E_0_ were decreased with the increment in moisture damage. Fatigue life and E_0_ can differentiate the moisture sensitivity of PA mixtures, and fatigue life was more sensitive to the moisture damage. Under long-term moisture damage, the moisture sensitivity of PA mixtures was still a concern.

(2) During the damage process, the residual ITS and E are decreased with the increasing loading cycles, and the largest decrement was found in the first 3000 loading cycles. PA mixtures tended to fail when the decrement in ITS and E exceeded 60% compared with the original. To extend the service life of PA mixtures, maintenance is needed when the decrement of ITS and E are greater than 60%.

(3) ITS and E, as well as the durability under the dry condition, are greater or better than that under the wet conditions during the moisture damage process. Moisture damage has a significant influence on the damage evolution of PA mixtures and has an acceleration function on the damage evolution.

(4) The durability of the PA mixture decreased with the increment in moisture damage and loading cycles. Under the wet condition of 8 d and after 12,000 loading cycles, the durability of PA mixtures could not satisfy the minimum requirement.

(5) The loading history of PA mixtures can be predicted by the damage factors of *λ_1_* and *λ_2_*. The damage factors and Cantabro loss could fit an exponential model under the dry condition. Under the wet condition, the durability damage evolution is more complicated due to the effects of moisture damage.

(6) In future research, the influences of binder properties, gradation, and additives on the moisture damage evolution need further investigations.

## Figures and Tables

**Table 1:** Properties of HVA.

Property	Value
Penetration (25 °C, 100 g, 5 s) (0.1 mm)	42
Penetration index	0.12
Ductility (5 cm/min, 5 °C) (cm)	30
Soft point (TR&B) (°C)	91
Recovery of elasticity (25 °C) (%)	92
Dynamic viscosity (60 °C) (Pa·S)	143,202
Density (25 °C) (g/cm^3^)	1.031

**Table 2:** Staged ITT fatigue test program.

Conditions	Loading Cycles	Number of Specimens
Dry	0	6
	3000	6
	6000	6
	9000	6
	12,000	6
Wet (2 d)	0	6
	3000	6
	6000	6
	9000	6
	12,000	6

## Data Availability

The data presented in this study are available on request from the correspondence author.
